# Contralateral Routing of Signal Disrupts Monaural Sound Localization

**DOI:** 10.3390/audiolres13040051

**Published:** 2023-08-03

**Authors:** Sebastian A. Ausili, Hillary A. Snapp

**Affiliations:** 1 Department of Biophysics, Donders Institute for Brain, Cognition and Behaviour, Radboud University, 6525 Nijmegen, The Netherlands; 2 Department of Otolaryngology, University of Miami, 1120 NW 14th Street, 5th Floor, Miami, FL 33136, USA

**Keywords:** contralateral routing of signal, localization, monaural listening, single-sided deafness, spatial hearing

## Abstract

Objectives: In the absence of binaural hearing, individuals with single-sided deafness can adapt to use monaural level and spectral cues to improve their spatial hearing abilities. Contralateral routing of signal is the most common form of rehabilitation for individuals with single-sided deafness. However, little is known about how these devices affect monaural localization cues, which single-sided deafness listeners may become reliant on. This study aimed to investigate the effects of contralateral routing of signal hearing aids on localization performance in azimuth and elevation under monaural listening conditions. Design: Localization was assessed in 10 normal hearing adults under three listening conditions: (1) normal hearing (NH), (2) unilateral plug (NH-plug), and (3) unilateral plug and CROS aided (NH-plug + CROS). Monaural hearing simulation was achieved by plugging the ear with E-A-Rsoft™ FX™ foam earplugs. Stimuli consisted of 150 ms high-pass noise bursts (3–20 kHz), presented in a random order from fifty locations spanning ±70° in the horizontal and ±30° in the vertical plane at 45, 55, and 65 dBA. Results: In the unilateral plugged listening condition, participants demonstrated good localization in elevation and a response bias in azimuth for signals directed at the open ear. A significant decrease in performance in elevation occurs with the contralateral routing of signal hearing device on, evidenced by significant reductions in response gain and low *r*^2^ value. Additionally, performance in azimuth is further reduced for contralateral routing of signal aided localization compared to the simulated unilateral hearing loss condition. Use of the contralateral routing of signal device also results in a reduction in promptness of the listener response and an increase in response variability. Conclusions: Results suggest contralateral routing of signal hearing aids disrupt monaural spectral and level cues, which leads to detriments in localization performance in both the horizontal and vertical dimensions. Increased reaction time and increasing variability in responses suggests localization is more effortful when wearing the contralateral rerouting of signal device.

## 1. Introduction

Sound source localization is dependent on the ability of the listener to extract interaural timing and level cues arriving at the two ears. In binaural hearing, integration of these cues plays a critical role in the ability to localize sound and separate speech from competing signals. Unilateral hearing loss abolishes binaural hearing cues. This significantly disrupts spatial hearing abilities and leads to impaired sound source localization [1]. One form of acquired unilateral hearing loss is single-sided deafness (SSD), which is characterized by severe-to-profound unilateral hearing loss. Interaural timing and level difference cues (ITD and ILDs) provide the primary cues for localization in the horizontal plane [2]. Poor spatial hearing experienced by SSD listeners is largely attributed to the loss of access to interaural timing and level cues arising from the asymmetry in hearing [3]. As a result, only physical cues such as the acoustic head shadow and monaural spectral pinnae cues remain available to SSD listeners [4]. Although less robust, these monaural cues may provide important information when binaural input is unavailable.

Evidence demonstrates that listeners can adapt to the monaural hearing condition over time, relying on level and spectral cues to improve their localization abilities [3]. Spectral pinna cues arise from the acoustic properties of the head and external ears and are important for vertical localization of sounds in elevation [5,6,7]. The concha acts as an acoustic resonator with the main spectral features produced by the direction-dependent filtering of the pinna for frequencies above 3–4 KHz [8]. Note that soundwaves in the 4–12 kHz range correspond approximately to wavelengths between 8.5 cm and 2.5 cm. Therefore, the observable spectral patterns of this cue are likely caused by geometrical features such as path, length, and cavities, which are integral components of the pinna [9,10,11]. For example, when a sound arrives from low-elevation angles the reflective path lengths through the helix-fossa of the pinnae are longer compared to when it enters from above the pinna. Due to the complexity of this cue, a proper characterization involves the inclusion of sound wave diffraction with head and pinna [12], as well as the effects of nontrivial resonances within the complex shapes of the 3D pinna cavities [13]. In the monaural hearing condition these cues are maintained for the intact ear. A growing body of research points to the ability of monaural listeners to reweight the physical cues to improve their spatial hearing abilities in the absence of treatment [14,15]. Spectral pinna are maintained under monaural hearing conditions [11,16,17,18]. Spectral cues are known to provide important information for localization in elevation and may also serve to help make judgments in azimuth [19,20].

Contralateral routing of signal (CROS) hearing aids are a common rehabilitation solution for individuals with SSD, serving to reroute the signal of interest from the impaired ear to the better-hearing ear [21]. Studies have shown that rerouting is successful at reducing some of the negative effects of the acoustic head shadow by amplifying sounds at the impaired side and can improve speech perception in noise for spatially separated signals [22,23,24]. However, tasks reliant on binaural hearing, such as localization, are not improved by rerouting the signal [22,23,24].

Despite offering this non-invasive treatment option for individuals with SSD, CROS technology has not gained widespread adoption and acceptance, primarily due to the known acoustic limitations [25]. It is possible that over time the reweighting [15] of monaural spectral cues reduces the initial handicap experienced by monaural listeners [25]. Moreover, it is also possible that those individuals who can reliably use monaural spectral cues to facilitate localization experience a disruption of these reweighted auditory cues with use of a CROS device [26]. Hearing devices that sit on the concha with a receiver placed in the ear canal interfere with the acoustics of the pinna, potentially altering spectral cues. The disruption of the subtle, yet essential monaural spectral cues, may be a limiting factor to CROS adoption and acceptance in monaural listeners [25].

Studies of CROS performance have focused almost exclusively on investigating the ability of these devices to improve localization performance in individuals with SSD [22,23,27,28,29]. Exploration of the potential detrimental effects of CROS in monaural localization are limited [26]. Moreover, prior studies of localization performance with CROS are largely restricted to the azimuthal plane. An investigation of the potential negative effect of CROS on localization performance by Pedley and Kitterick (2017) suggests that CROS does restrict the use of monaural cues for localization in azimuth. These findings raise important questions about how CROS affects listeners’ ability to use monaural cues for spatial hearing. The effect of CROS on localization performance in azimuth and elevation for monaural listeners has not been established. Yet, removal of spectral pinna cues has been shown to disrupt vertical localization [11,30]. Studies suggest little effect of in-the-ear or behind-the-ear hearing aids on vertical localization in traditional hearing aid users [31,32]. Although, this is not surprising given the limited ability to make use of spectral cues due to the presence of high-frequency hearing loss [19] and limited bandwidths of hearing aids to adequately restore access to cues above 4000 Hz [33]. The impact of CROS hearing aids on localization in monaural listeners, where good high-frequency hearing in the normal ear provides listeners with access to monaural spectral cues, is less understood. While the effects of CROS devices on localization of sounds in the azimuthal plane have been investigated, little is known about their impact on elevation localization and the potential disruption of monaural cues. The current study aims to address this gap by exploring the effect of CROS on monaural sound localization in both azimuth and elevation under simulated monaural listening conditions.

## 2. Methods

### 2.1. Participants

Ten normal hearing adults (mean age 27 ± 5 years, range 22–37 years) participated in this study. All participants had normal hearing bilaterally as determined by air conduction hearing thresholds of <20 dB HL across the standard audiometric test frequencies of 250–8000 Hz, and no prior history of hearing impairment. All participants were naïve to the experimental procedures. All experimental procedures have been approved by the local ethics committee of the Faculty of Social Sciences of the Radboud University (ECSW 2016-2208-41).

### 2.2. Experimental Setup and Stimuli

**Figure 1:**
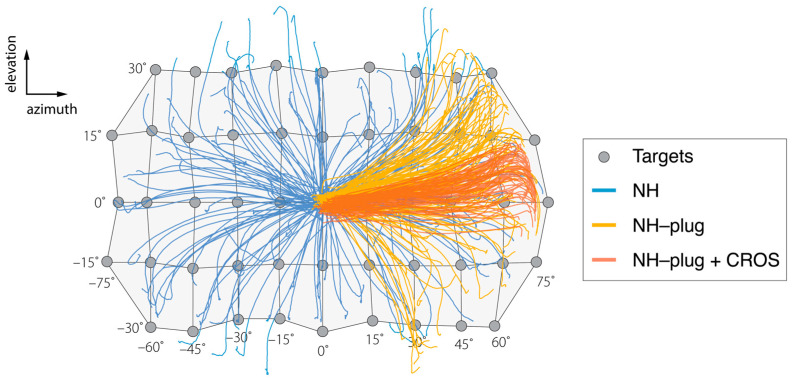
Results of the head-tracking for localization of targets presented in azimuth and elevation are presented over a schematic of the target matrix for the sound localization experiment. Negative values indicate stimuli presented to the left (plugged) side. The grey circles indicate each source location in azimuth and elevation. Blue lines show the orientation behavior of a representative listener (P3) in the NH condition, where all targets are well-localized. (P3). Yellow lines represent responses under the NH-plug condition. Left ear plugging results in biased responses to the right hemifield while sources in elevation remain well-localized. Responses in red represent NH-plug + CROS condition and demonstrate extreme bias to the open ear in azimuth and further disruption of vertical localization.

Sound localization measurements were performed in a completely darkened sound-isolated chamber [34,35]. Stimuli were presented in a random order from 50 locations in a plane spanning −70° (left) to +70° (right) in the horizontal and ±30° in the vertical plane, located 1.3 m from the listener (Figure 1). All sounds consisted of 150 ms Gaussian white noise bursts, with 5 ms sine-squared onset and offset ramps, presented randomly at 45, 55 and 65 decibels (dB), A-weighted (dBA), totaling 150 trials per listening condition. Stimuli were also high-pass filtered (3–20 kHz), focusing on high-frequency content of spatial cues (i.e., ILDs and monaural spectral cues).

During the task, participants sat comfortably in a chair and were instructed to fixate a head-mounted light emitting diode (LED) towards the perceived sound location via a head movement. Horizontal and vertical head movements were recorded with the magnetic search coil technique to measure the head rotation of the subject in response to the stimuli. The presentation of stimuli and data acquisition were implemented using TDT3 hardware (Tucker–Davis Technologies, Alachua, FL, USA) and controlled with a custom-made software based in MATLAB (version R2015a, The MathWorks Inc., Natick, MA, USA).

### 2.3. Sound Localization Paradigm

Prior to each trial, subjects had to fixate to a visual target presented straight ahead at 0° azimuth (α) and 0° elevation (ε) to ensure proper head orientation. After pressing a button, the fixation LED was turned off within 100 to 300 ms, and the sound stimulus was presented 200 ms later. The subject was asked to orient the head-fixed laser dot as fast and as accurately as possible to the perceived sound location. The acquisition time for head movements was 1.5 s, after which the central fixation LED was switched on to start the next trial. To familiarize the subject with the experimental procedures, a practice session of 15 trials was carried out before the actual experiment. No feedback was given about actual localization performance.

### 2.4. Listening Conditions

Sound localization experiments were conducted for three listening conditions: (a) normal hearing (NH), (b) unilateral plug (NH-plug), and (c) unilateral plug and CROS aided (NH-plug + CROS). All participants were plugged in the left ear for the NH-Plug and NH-plug + CROS conditions with E-A-Rsoft™ FX™ foam earplugs (3 M™ New Zealand Pty, Limited), which attenuated sounds above 3000 Hz by at least 30 dB. The three experimental conditions were performed in succession in a single experimental session lasting approximately 40 min. Note that although the unilateral plugged conditions significantly perturbed binaural hearing cues, complete monaurality could not be achieved. Some remnant ITD and ILD cues may have been available to listeners under plugged listening conditions, particularly at high presentation levels (65-dBA). When occluding one ear in normal-hearing listeners, monaural level and spectral-pinnae cues are enhanced sound localization cues [1,36,37]. Monaural plugging mostly induces ILD cues resulting in a large bias in azimuth towards the open ear while localization in elevation is abolished on the occluded side [37]. Moreover, monaurally occluded normal-hearing listeners maintain the use of high-frequency spectral cues on the open ear and therefore are a suitable model to study potential disruption of these cues with CROS devices [26].

All experiments were performed using a behind-the-ear Phonak Audeo V50^®^ CROS open fit hearing aid system. This hearing aid consists of a microphone placed on the superior portion of the pinna of the impaired ear, which wirelessly transmits acoustic information (130–6000 Hz) to a receiver hearing aid worn in the normal ear. Devices were fitted using NAL-NL prescriptive targets [38] with hearing thresholds set to 15 dB HL across the frequencies, and microphones set to omnidirectional mode. Consistent with standard fitting practices in CROS users, listeners were fit with a non-occluding, flexible 6 mm open dome. During testing conditions, volume control was inactive to ensure prescriptive targets were maintained.

### 2.5. Data Analysis

To evaluate sound localization stimulus–response relationship in azimuth and elevation, the optimal regression line was determined by minimizing the sum-squared error through the data points as follows:

$$\alpha_{R}=a+b\;\cdot \;\alpha_{T}\;\mathrm{and}\;\varepsilon_{R}=c+d\;\cdot \;\varepsilon_{T}$$

where α is azimuth, ε is elevation, $\alpha_{R}$ and $\varepsilon_{R}$ are the azimuth and elevation response components, and $\alpha_{T}$ and $\varepsilon_{T}$ reflect the target coordinates of the stimulus in degrees. Fit parameters, *a* and *c*, is the offset (in degrees) or listener’s bias, whereas *b* and *d* are the slopes, or gains (dimensionless) of the azimuth and elevation responses, respectively. We also computed the coefficient of determination (*r*^2^), or goodness of fit, for all linear fits. Note that a perfect localization response should yield gains of 1.0 and offsets of 0.0° and a high coefficient of determination of 1.0. Moreover, we also study the elevation sound localization performance in more detail to observe differences along the azimuth domain. Considering the speaker’s arrangement, the horizontal range was divided into nine non-overlapping contiguous 15°-wide windows. On each 15° section a regression line was computed from the elevation target–response relationship [34].

Reaction times (in milliseconds) were measured by taking the difference between stimulus onset and head movement onset. The data were transformed to its reciprocal, known as the response promptness (in s^−1^). This way, the distribution of data follows a nearly Gaussian distribution (Carpenter et al., 1995), from which we determined the mean and its standard error.

A factorial analysis of variance (ANOVA) was used to assess differences across localization gain, bias, and coefficients of determination outcomes (dependent variables) for all listening conditions and presentation levels (factors). A multiple comparison of the ANOVA estimates was computed, with Bonferroni correction, and post hoc analysis to determine further significant differences. A probability value of *p* < 0.05 was taken as the level of significance. All data analysis was performed in MATLAB’s Statistics toolbox (version R2020b, The MathWorks, Natick, MA, USA).

## 3. Results

### 3.1. Sound Localization Performance

Figure 1 shows a representative example of the target response behavior of a single participant in two dimensions (azimuth and elevation), across the three experimental conditions. A grid is used to show the target coordinates in the horizontal (±70°) and vertical (±30°) planes from which the stimuli were presented. In the NH condition (blue), responses reflect the target matrix, covering the entire target range. Localization accuracy of the head-orienting response is observed in both the horizontal and vertical dimensions. As expected, the NH-plug condition (yellow) shows a clear response bias towards the open ear (α > 0°). Yet, vertical sound localization responses are maintained across the elevation target range on the open side. Head-orienting responses in this condition spanned −30° < ε > +30°, indicating preservation of vertical localization. In the NH-plug + CROS condition (red), localization in azimuth and elevation are visibly disrupted by the CROS device. Responses are compressed in both the vertical and horizontal dimensions of the target range, yielding stronger horizontal bias and a deficiency in elevation over the two-dimensional space compared to the NH-plug and NH-plug + CROS conditions.

**Figure 2:**
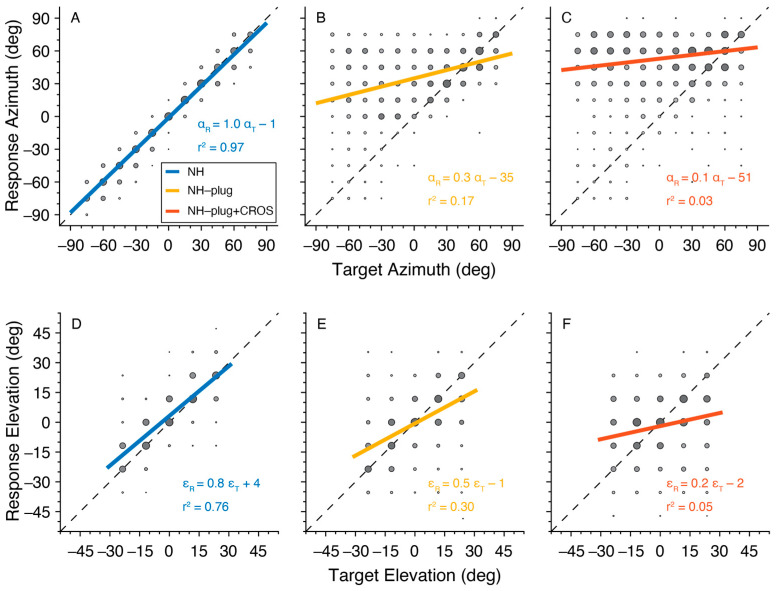
Target-response plots are presented for each listening condition pooled across all participants. NH is presented in blue (**A**,**D**), NH-Plug in yellow (**B**,**E**) and NH-plug + CROS in red (**C**,**F**). Top panel (**A**–**C**): responses in azimuth; bottom panel (**D**–**F**): responses in elevation. Larger circles indicate larger number or responses at the associated target-response location. The regression line represents the best linear fit of the pooled data. Negative target−azimuth values indicate stimuli presented to the left (plugged) side.

Target-response plots presenting the localization performance of listeners in azimuth and elevation for the three listening conditions are presented in Figure 2. Here, pooled responses for all 10 participants are depicted by gray circles with larger marks indicating more responses at the associated target location. A typical response pattern under NH conditions is observed. In azimuth, overall sound localization patterns yielded a high degree of accuracy evidenced by gains close to 1, lack of bias, and *r*^2^ close to 1 (Figure 2A). When listeners were unilaterally plugged (NH-plug), results in azimuth revealed a strong response bias to the open ear (α = −35°), and a decrease in localization gain is observed (Figure 2B). The target-response plots for localization performance in elevation suggests less of an effect of the plug on gain or response bias, however, a high overall response variability is reflected by a low *r*^2^ (Figure 2E). The addition of the CROS hearing aid negatively affected sound localization in both the vertical and horizontal dimensions. In the NH-plug + CROS condition, gain was further reduced and an increase in response bias towards the open ear was observed in azimuth, with the *r*^2^ decreasing to nearly 0 (Figure 2C). Moreover, a clear disruption of localization performance in elevation also occurs when wearing the CROS hearing aid. Figure 2F shows that elevation gain is significantly reduced in the NH-plug + CROS condition when compared to the plug alone (Figure 2E).

**Figure 3:**
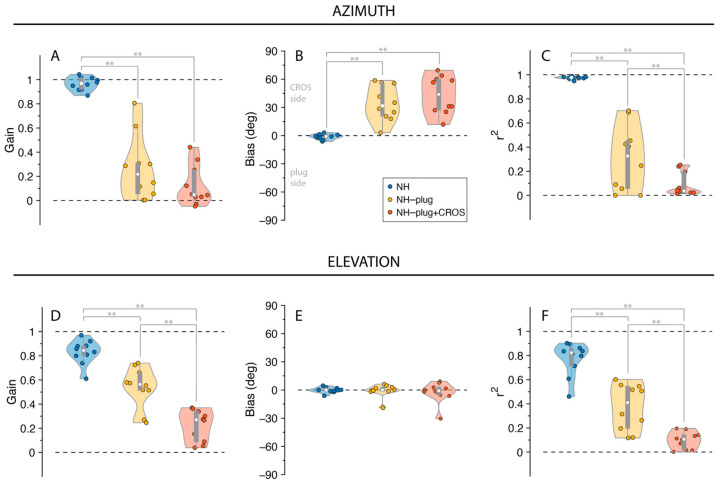
Gain (**A**,**D**), bias (**B**,**E**), and *r*^2^ (**C**,**F**) values are presented for responses in azimuth and elevation. NH is presented in blue, NH-Plug in yellow and NH-plug + CROS in red. Top panel (**A**–**C**): responses in azimuth; bottom panel (**D**–**F**): responses in elevation. Violin plots present each participant performance (colored circles) with the 25th and 75th percentiles indicated by the gray bars and the median indicated by the white circle. Significance of the effect of condition are indicated with ** when *p* < 0.001.

The distribution of listeners’ individually fitted (i.e., unpooled) response gains, biases and *r*^2^ values across all experimental conditions is presented in Figure 3. A Factorial ANOVA was conducted to compare the main effects of listening condition and stimulus level and the interaction effect between listening condition and stimulus level on localization. For responses in azimuth, analyses revealed a significant main effect of listening condition on localization gain in the azimuthal plane with an F ratio of (F_(α,gain)_[2,81] = 152.67, *p* < 0.001), localization bias (F_(α,bias)_[2,81] = 63.75, *p* < 0.001), and *r*^2^ (F_(α,r2)_[2,81] = 166.35, *p* < 0.001). The post hoc comparisons revealed that α-gain, α-bias, and α-*r*^2^ under NH-plug and NH-plug + CROS conditions were significantly poorer than the NH condition. Note that the goodness of fit of the NH-plug + CROS condition decreased significantly compared to NH-plug, evidencing poor localization (Figure 3C). Responses in elevation yielded significant main effects for gain (F_(ε,gain)_[2,81] = 99.44, *p* < 0.001) and *r*^2^ (F_(ε,r2)_[2,81] = 133.44, *p* < 0.001). Post hoc comparisons revealed significant differences between all listening conditions, reflected by a systematic decrease in ε-gain and ε-*r*^2^ (Figure 3D,F). There was no effect of condition on bias in elevation (F_(ε,bias)_[2,81] = 1.44, *p* = 0.25; Figure 3E).

Stimulus presentation level did not influence localization performance in either the azimuthal or vertical dimensions. Analyses revealed no effect of stimulus level (45, 55 and 65 dBA) on localization gain (F_(α)_[2,81] = 1.25, *p* = 0.29; F_(ε)_[2,81] = 0.45, *p* = 0.64), localization bias (F_(α)_[2,81] = 0.09, *p* = 0.92; F_(ε)_[2,81] = 1.47, *p* = 0.24), or *r*^2^ (F_(α)_[2,81] = 0.89, *p* = 0.41; F_(ε)_[2,81] = 1.45, *p* = 0.24). Moreover, no interaction was observed between listening conditions and presentation level for gain (F_(α)_[4,81] = 0.73, *p* = 0.58; F_(ε)_[4,81] = 0.73, *p* = 0.58) or bias (F_(α)_[4,81] = 0.07, *p* = 0.99; F_(ε)_[4,81] = 0.08, *p* = 0.99) or *r*^2^ (F_(α)_[4,81] = 0.27, *p* = 0.90; F_(ε)_[4,81] = 0.27, *p* = 0.89) in either the horizontal or vertical dimensions.

#### The Influence of Spectral Cues on Localization Performance in Azimuth

**Figure 4:**
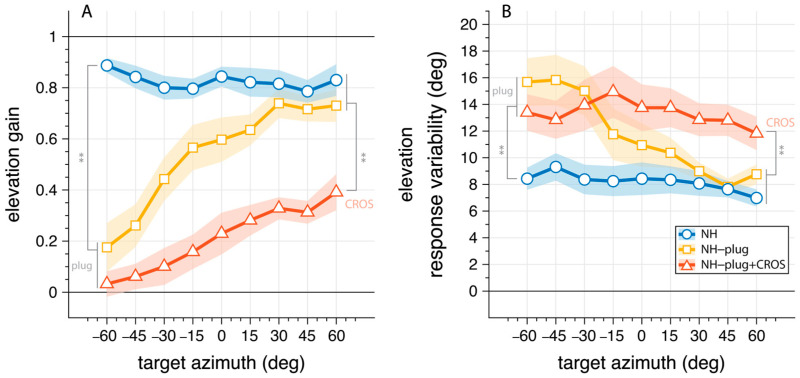
Elevation gain (**A**) and response variability (**B**) as a function of target azimuth. The connected markers denote the binned mean across listeners with the shaded area indicating the standard error. The NH condition is presented in blue; NH-plug in yellow; NH-plug + CROS in red. All significant effects are depicted with ** where *p* < 0.001. Negative values indicate stimuli presented to the left (plugged) side.

To examine the effects of asymmetric hearing on the use of monaural cues, we analyzed the vertical sound localization performance as a function of stimulus azimuth (Figure 4). Here, elevation gain (Figure 4A) and response variability (Figure 4B) are pooled and presented as a function of azimuth target location to depict differences in accuracy and response variability for signals presented at the open ear versus the plugged ear. In the NH condition listeners correctly localize vertical sounds independent of target azimuth (Figure 4A, blue). The mean elevation gain for the NH condition was >0.8 across the entire azimuth range. Response variability in the NH condition was 8.2°.

Under plugged hearing conditions (Figure 4, yellow, red), vertical localization performance is strongly diminished at the plugged side (α < 0°). This is clearly reflected on the plugged side (α = −60°), where both monaural conditions are significantly different from NH performance in gain (F_(ε,gain,α=−60°)_[2,27] = 50.96, *p* < 0.001) and response variability (F_(ε,var,α=−60°)_[2,27] = 7.27, *p* = 0.003). Post hoc comparisons showed no difference between NH-plug and NH-plug + CROS on the far-left side where listeners were plugged. On the open hearing side (right), there was a main effect of conditions towards the periphery of the hemifield for both gain (F_(ε,gain,α=60°)_[2,27] = 12.33, *p* < 0.001) and variability (F_(ε,var,α=60°)_[2,27] = 7.13, *p* = 0.003). Post hoc comparisons indicated performance with the CROS device was significantly poorer than the other listening conditions (Figure 4). Since the open ear remains intact in NH and NH-plug listening, granting access to spectral pinna cues, performance yielded high elevation gains (means: NH_(ε,gain,α≥30°)_ = 0.8; NH-plug_(ε,gain,α≥30°)_ = 0.7) and low response variation (means: NH_(ε,var,α≥30°)_ = 7.5°; NH-plug_(ε,var,α≥30°)_ = 8.5°). However, this performance is significantly impaired when the CROS device is applied (Figure 4, red), evidenced by decreased elevation gains (mean: NH-plug + CROS_(ε,gain,α≥30°)_ = 0.3) and increased response variability (mean: NH-plug + CROS _(ε,var,α≥30°)_ = 12.5). The fact that elevation gain is maintained at the open ear but significantly diminished when the CROS is applied which is concomitant with a significant increase in response variability, provides support that monaural spectral cues are disrupted by the use of a CROS device.

### 3.2. Sound Localization Promptness

**Figure 5:**
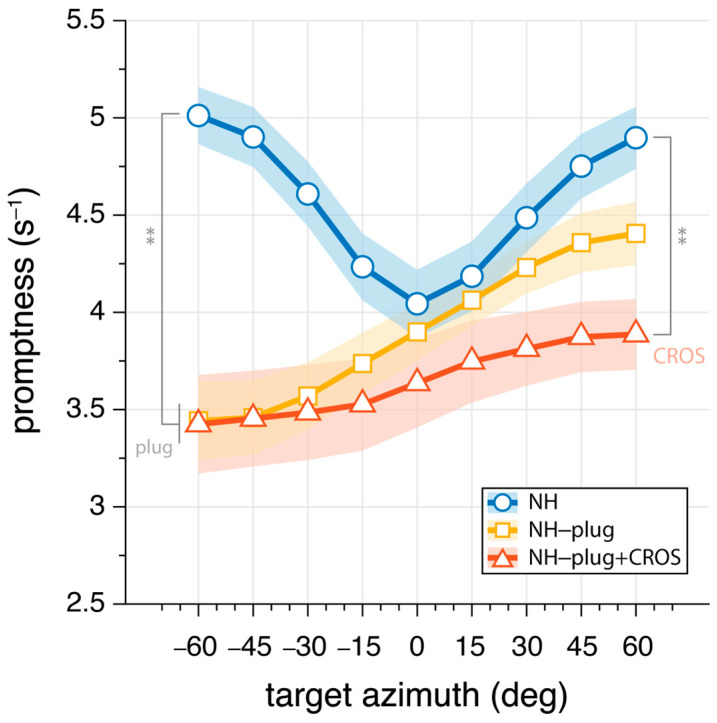
Mean promptness (inverse of reaction time) as a function of target azimuth. Greater values indicate increase promptness of the response. Markers connected by lines denote binned mean promptness across listeners, whereas the shaded area denotes the standard error across listeners. NH conditions are presented in blue, NH-plug in yellow, and NH-plug + CROS in red. All significant differences are indicated with ** for *p* < 0.001.

To further evaluate the effects of CROS devices on the use of monaural cues, the promptness of responses across azimuth was analyzed. Response promptness to the auditory stimuli is presented for all participants in Figure 5. As with the elevation gain and variability, promptness is plotted as a function of target azimuth location, analyzed in 20° azimuth windows and pooled across levels. In the NH condition (blue), responses were faster than monaural hearing conditions throughout the entire azimuth range (mean: NH_P_ = 4.6 s^−1^). NH-plug and NH-plug + CROS response patterns show a significant decrease on the plugged side (F_(P,α=−60°)_[2,27] = 19.87, *p* < 0.001), reaching 3.4 s^−1^ on average. From the plugged to the open hearing side, there is a systematic increase in promptness (faster responses). Maximum promptness is reached at α = 60° for all conditions, with a reduction in peak promptness of the response for the NH-plug + CROS (mean: NH-plug + CROS_(P,α=60°)_ = 3.9 s^−1^) compared to the plug alone (mean: NH-plug_(P,α=60°)_ = 4.4 s^−1^). There was a main effect of listening condition at α = +60° (F_(P,α=60°)_[2,27] = 9.08, *p* < 0.001), and although Figure 5 shows that promptness increased with greater perturbation of normal hearing, post hoc analysis revealed only a significant difference between the NH and NH-plug + CROS listening conditions.

## 4. Discussion

Due to the loss of binaural hearing, SSD listeners suffer from poor speech perception in noisy environments and reduced sound localization. In more recent previous studies on the benefits and limitations of rehabilitative devices in monaural listeners have been limited to localization in azimuth. This approach neglects to take into account the role of spectral pinna cues in vertical sound localization. Monaural localization is well studied, but studies on the effects of ear level devices (i.e., hearing aids worn behind and/or in the ear) on monaural localization performance in monaural listeners are lacking. Research has shown that listeners can learn to reweight spatial hearing cues under monaural listening conditions to improve their spatial hearing abilities [15,39,40]. The most widely accessible non-surgical treatment for individuals with SSD is the CROS hearing aid, which may serve to impede access to the cues monaural listeners become reliant on. The results herein demonstrate that the application of a CROS hearing aid significantly disrupts the monaural spectral cues that give rise to localization in elevation. The acute pattern of localization changes in azimuth and elevation presented here demonstrates that monaural cues used to make judgments in space are negatively affected by the use of a CROS device.

The pinna performs direction-dependent filtering for high-frequency signals essential for localization in elevation [41]. Here, a decrease in both horizontal and vertical localization performance is observed (Figure 2 and Figure 3) under monaural hearing conditions (NH-plug), evidenced by a decrease in response gains and localization precision (*r*^2^) compared to the NH condition. Although, a closer review of the data (Figure 4) shows that spectral cues at the open ear are preserved for vertical localization, as is consistent with that observed in untreated SSD listeners [42]. Good localization performance in elevation is maintained on the open side, showing that spectral cues remain available for the non-occluded ear but are abolished on the side of the plug (Figure 1). Consistent with previous work [37], these findings demonstrate that even under acute monaural listening situations, spectral cues on the side of the intact ear are immediately recruited for azimuth localization when the contralateral ear is occluded.

In the NH-plug condition listeners had better localization in azimuth on the side of the open ear compared to the plugged ear (Figure 2). Analysis of localization performance by stimulus level found no effect on gain, bias, or *r*^2^ demonstrating that listeners did not rely on level cues to inform sound source localization, and it is unlikely that listeners were able to rely significantly on remnant binaural cues given the presentation levels, stimulus frequency range, and attenuation caused by the plug. Rather, this observation indicates that the listeners leveraged monaural spectral cues to make judgments for localization in azimuth. Unilateral plugs are a conventional method to monauralize listeners, but cannot be expected to produce a total sound isolation [37] as would be the case with a true SSD listener. However, the strong azimuth bias observed in our data suggests that the combination of unilateral plugging with high-pass stimuli was effective in monauralizing listeners for the proposed experiments.

When the CROS hearing aid was applied, the good vertical localization at the open ear was obliterated (Figure 3). This is evidenced by poor response gains, low coefficient of determination (*r*^2^), increased response variability, and slowed response rates (i.e., promptness, Figure 4). Decreased performance in azimuth is also observed when the CROS hearing aid is applied. Pedley and Kitterick (2017) also found the CROS device had a detrimental effect on the ability to localize sounds in azimuth. In their study, monaural noise recordings were made in the ear canal with and without a CROS hearing aid and filtered to manipulate spectral and level cues. The signals were delivered via earphones to participants who were asked to determine the location of the signal in azimuth using a three-alternative forced-choice paradigm (right, left, or in front of the listener). Not observed in the study by Pedley and Kitterick (2017) are our findings of decreased localization abilities in azimuth at the open ear side. Potentially, the three-alternative forced-choice spatial discrimination task used in their study was not sensitive enough to detect the observed effects in azimuth on the open ear side. Their work also suggested that monaural level (head shadow) cues can no longer be used by the listener to help locate sounds. Decreased performance in elevation at the open ear in our results indicate that the spectral pinna cues are perturbed by the CROS device. Compared to conditions where the open ear is unmodified (NH, NH-plug), response to targets on the open ear side suggests that the presence of the device on the ear and in the ear canal itself is altering the monaural spectral cue. Moreover, given there was no main effect of sound level in the present study, observed changes in localization performance with the addition of the CROS device is likely caused by its disruption of spectral cues rather than monaural level cues (Figure 3 and Figure 4). This is underscored by the lack of level effect in the NH-plug + CROS condition where localization in azimuth is further disrupted. No effect of level in the NH-plug + CROS condition excludes a potential contribution of head shadow to the present results.

As others have shown, under acute monauralization, listeners present with a biased localization response to the open ear side but maintain good vertical localization [18,40,41]. Further, listeners demonstrate an immediate upweighting of spectral cues to facilitate localization in azimuth when normal binaural hearing is perturbed [26]. These findings support that spectral cues in azimuth and elevation are not entirely independent. Hebrank and Wright found that spectral pinna cues facilitate localization in azimuth [41]. Depending on the direction of the sound source, the pinna will enhance some components of the frequency spectrum and attenuate others [5]. Even in the monaural hearing condition these cues can be easily learned and applied to improve ones’ ability to locate sound sources in azimuth [6]. This is of particular importance when considering the benefits and limitations of rehabilitative devices or treatments for individuals with SSD. It is well known that CROS devices cannot improve localization, but the question of whether CROS device can negatively affect monaural localization abilities that may be acquired over time by SSD listeners is overlooked. Figure 4 (yellow) shows strong elevation gain in the open ear side when in the NH-plug condition, demonstrating the immediate ability of listeners to use monaural cues to facilitate localization in the horizontal as well as the vertical planes. Conversely, elevation response gains are low on the side of the plugged ear and a significant increase in response variability is observed compared to the NH condition for signals presented to the plugged side. As the signal is moved in azimuth toward the open ear (right), elevation response gain increases with an associated decrease in response variability. Accordingly, the reaction time also decreases for signals presented to the open ear side compared to the plugged side. Collectively, these results demonstrate that response variability increases concurrent with poorer localization performance and increased reaction times at the plugged side.

The effect of the CROS device on localization performance in the open ear is far more pronounced. With the addition of a CROS device in the intact ear (Figure 4, red), not only is vertical localization severely perturbed, but spectral cues are disrupted further compromising any residual localization ability. Addition of the CROS device resulted in a significant increase in response variability and a decrease in promptness of the response, suggesting listeners were less certain in their localization responses (Figure 5). Eliminating access to reliable cues results in ambiguity of the signal, which is reflected in the response behavior. Reaction times are known to increase with stimulus uncertainty [43] and task complexity [44], and have been used as a measure of listening effort [43,44,45]. Here, promptness of the response serves as an index of performance. As observed by others, there is a decrease in promptness of the response that is associated with reduced performance [34,45], suggesting more effort is required for the localization task. As shown by others, the eccentricity effect where a decrease in promptness occurs in the central azimuth region occurred in the NH condition [46,47,48]. Our data shows the lack of this effect in the monaural conditions, which is consistent with other spatial hearing studies with asymmetric listening conditions [34].

Our findings offer some insight into those monaural listeners who do adapt and become reliant on monaural spectral cues, in particular young listeners with normal high-frequency hearing who reject CROS as a treatment solution for SSD. This may be of value when determining candidacy for various treatment solutions for individuals with SSD. It is possible that the hearing in noise and sound awareness benefits of CROS [24] do not outweigh unrecognized detriments incurred by the use of an ear level device on monaural localization in such listeners. Cochlear implants, for example, are increasingly prevalent in the management of SSD due to the potential to provide listeners some binaural hearing benefit. In those individuals with contralaterally normal hearing, a cochlear implant does not interfere with the intact ear, thereby preserving its natural spatial cues. Although, not all monaural listeners learn to reweight monaural spectral cues to improve their localization abilities. Agterberg et al. (2014) showed that monaural localization performance was poorer in adults with hearing loss at 8000 Hz, indicating these listeners could not make use of spectral pinna cues to improve performance. Conversely, a listener who cannot make use of monaural spectral cues (i.e., those with high-frequency hearing loss in the better ear) may be more likely to perceive benefit from the reduction of the acoustic head shadow offered by CROS hearing aid systems. It should be noted that the responses measured here were in normal-hearing listeners under acute unilateral plugging. It is possible that listeners could adapt to the CROS-aided listening condition thereby improving performance with time, as has been shown in unaided monaural listeners. Specifically, CROS introduces a novel unreliable spectrum at the good ear that could potentially be relearned over time or with training. Although, the amplification of the device may impact the reliability, negatively affecting any adaptation or training effects. Future studies will focus on answering these questions in established CROS device users.

## 5. Conclusions

Normal pinna function is distorted by the CROS hearing aid which leads to detriments in localization performance in both the horizontal and vertical dimensions. Sounds are mostly perceived towards the extreme side of the functional ear on azimuth and monaural spectral cues are functionally perturbed for localization in both elevation and azimuth. Furthermore, this drop in performance comes with higher effort and yields higher uncertainty of sound location. For those monaural listeners who successfully adapt to the use of such monaural spectral cues, this may contribute to failure to adopt CROS technology. Collectively, these results demonstrate that the CROS hearing aid diminishes the access to monaural spectral cues and suggests that reweighting of monaural spectral cues may not be possible with the use of an ear level device on the better-hearing ear.

## Data Availability

Data can be made available upon request, subject to the discretion of the authors.
